# Synthesis of Nylon 6/Modified Carbon Black Nanocomposites for Application in Uric Acid Adsorption

**DOI:** 10.3390/ma13225173

**Published:** 2020-11-17

**Authors:** Marlene Andrade-Guel, Carlos A. Ávila-Orta, Gregorio Cadenas-Pliego, Christian J. Cabello-Alvarado, Marissa Pérez-Alvarez, Pamela Reyes-Rodríguez, Fawad Inam, Dora A. Cortés-Hernández, Zoe V. Quiñones-Jurado

**Affiliations:** 1 Center for Research in Applied Chemistry (CIQA), Saltillo, Coahuila 25294, Mexico; marlene.andrade@ciqa.edu.mx (M.A.-G.); carlos.avila@ciqa.edu.mx (C.A.Á.-O.); pamarissa@hotmail.com (M.P.-A.); pamela.reyes@ciqa.edu.mx (P.R.-R.); 2 CONACYT Research Fellow-Research and Innovation Consortium of the State of Tlaxcala, Tlaxcala C.P. 90000, Mexico; 3 Department of Engineering and Construction, University of East London, London E16 2RD, UK; 4 Center for Research and Advanced Studies of the National Polytechnic Institute (CINVESTAV) Saltillo Unit. Av. Industria Metalúrgica #1062 Parque Industrial Saltillo-Ramos Arizpe, Saltillo 25900, Mexico; dora.cortes@cinvestav.edu.mx; 5 Faculty of Chemical Sciences, Durango State Juárez University, Durango C.P. 34120, Mexico; zoevineth@gmail.com

**Keywords:** carbon black, hemolysis, adsorption

## Abstract

High uric acid levels cause different clinic conditions. One of them is hyperuricemia, which leads to kidney damage. A solution for eliminating uric acid in the blood is by hemodialysis, which is performed using nanocomposite membranes. In this work, Nylon 6 nanocomposites were synthesized with modified carbon black (MCB), which were considered candidate materials for hemodialysis membranes. The modification of carbon black was made with citric acid using the variable-frequency ultrasound method. The new MCB was characterized by Fourier transform infrared spectroscopy (FTIR), thermogravimetric analysis (TGA), X-ray diffraction (XRD), transmission electron microscopy (TEM), and dispersion tests. Nylon 6/MCB nanocomposites were processed using the ultrasound-assisted melt-extrusion method to improve the dispersion procedure of the nanoparticles. The Nylon 6/MCB nanocomposites were characterized by FTIR, TGA, and differential scanning calorimetry (DSC). These were assessed for the absorption of toxins and hemocompatibility. MBC and nanocomposites showed excellent uric acid removal (78–82%) and hemocompatibility (1.6–1.8%). These results suggest that Nylon 6/MCB nanocomposites with low loading percentages can be used on a large scale without compatibility problems with blood.

## 1. Introduction

Cases of hyperuricemia have increased in recent years [1,2]. This is linked to people’s lifestyle and the consumption of high-sugar food. It is sometimes a consequence of fructose metabolism in different foods or alcohol, resulting in an increase in uremic acid and leading to hyperuricemia [3]. Hyperuricemia can cause conditions such as hypertension, cardiovascular disease, and kidney damage [4]. Uric acid is a final metabolite of purines in humans, and there are two routes through which purine can enter the human body: through oral intake or biosynthesis. Oral intake refers to major uric acid consumption, which tends to accumulate in some body parts and reacts through other types of composites produced by the body. Uric acid excess inhibits the generation of nitric oxide, a vasodilator substance, and conduces endothelial dysfunction, which is one of the factors involved in the development of arteriosclerosis [5,6]. Another illness related to high levels of uric acid is hypertension, which results from animal models and suggests a two-phase mechanism for the development of hyperuricemia hypertension. Uric acid induces acute vasoconstriction by activating the renin-angiotensin system, followed by uric acid absorption into vascular smooth muscle cells leading to cell proliferation and secondary arteriosclerosis. Available data suggest that uric acid is potentially the cause of some cases of hypertension in people of a younger age [7]. There is strong evidence that relates uric acid to renal disease [4,8]. During the process of hyperuricemia-induced kidney injury, uric acid crystal deposition is involved in a common pathway that triggers kidney injury and inflammation. Crystalline kidney injury is accepted in urinary tract obstruction [9]. Uric acid is a uremic toxin that presents the greatest adsorption problem due to its tautomeric equilibrium. Carbon-based nanostructures, such as graphene nanoplatelets (GNPs) with basic and acid modifications, are excellent for adsorbing this type of toxin [10,11]. Carbon black (CB) is used as an adsorbent because it has various properties such as a significantly large surface area, high microporosity, and low cost compared to other carbonaceous materials of nanometric size [12]. To clean the blood of uric acid, dialyzers are normally used. Studies have shown that the uric acid removal ratio of a single group is 60–70%. For example, with high-flow dialyzers, approximately 61–64% of uric acid is removed from the body, whereas with low-flow dialyzers, only the toxin is eliminated between 50% and 55% [13]. Dialyzers are made with cellulose membranes [14] and other polymers such as Nylon-6 due to their excellent chemical and thermal resistance and wettability [15]. The use of Nylon 6/CB nanocomposites can be an excellent alternative for use in the removal of uremic toxins, as these nanocomposites combine excellent physicochemical properties and low cost.

Dispersion is a key factor in the making of polymer nanocomposites for the nanoparticles (NPs) to be properly distributed in the polymer matrix [16]. Melt-extrusion is considered an efficient technology in this field, with particular advantages over solution polymerization processes and in situ polymerization. Melt-extrusion is a process of converting raw material into a product of uniform shape and density by forcing it through dye under controlled conditions [17]. The dispersion of NPs in polymers when synthesizing polymer nanocomposites depends on the process and type of extruder. The use of the ultrasound-assisted melt-extrusion method achieves good dispersion of NPs in the polymer matrix. Ultrasonic waves help to separate agglomerated particles or may even be able to fracture the particles when they are tightly bound as aggregates [18]. The dispersion mechanism depends on the conditions of the ultrasonic treatment. Conventional fixed frequency ultrasonic application has been used for more than 20 years [19,20,21]. However, due to the high viscosity of molten polymers, this mechanism is not sufficient for improving particle dispersion and deagglomeration. Another mechanism has been recently proposed to explain how ultrasonic waves can help reduce the size of NPs agglomerates during the extrusion process. This mechanism involves the vibratory movement of polymer chains due to their characteristic resonance frequency, which, in turn, is responsible for the break of particle agglomerates. With the use of ultrasonic waves of different frequencies and amplitudes applied in the form of dynamic scanning in a range of frequencies, several chain lengths can be disturbed [22]. The incorporation of NPs into the Nylon 6 matrix affects the physicochemical and mechanical properties of nanocomposites. Nylon 6 nanocomposites with NPs of different natures have been reported [21,23,24,25,26,27].

The objective of this work was (1) to modify CB with citric acid (renewable compound) using variable-frequency ultrasound at different reaction times; (2) study the adsorption of uric acid to later incorporate it into a polymeric matrix of Nylon 6 using the ultrasound-assisted melt-extrusion method; (3) and evaluate the absorption properties in the removal of uric acid.

## 2. Materials and Methods

### 2.1. Materials

VULCAN XC-72 CB (CABOT, Altamira, TAM, Mexico) with a diameter of 15 nm and a purity of 99% was used. The citric acid used has a purity of 99% (Sigma Aldrich, Saint Louis, MO, USA), and distiller water with a pH of 7 was used for all concentrated aqueous solutions.

For the preparation of nanocomposites, we employed a polymeric Nylon 6 matrix commercially known as Zytel®7301 NC010 (DuPont, Wilmington, DE, USA). In accordance with the specification sheet, Nylon 6 complies with physical characteristics to manufacture fibers or fabrics. Some of its characteristics include a density of 1.13 g/cm^3^, a melting point analogous to 230 °C, a molecular weight by weight (Mw, calculated by gel permeation chromatography (GPC)) of 35,610 g/mol, and a 1.7 polydispersity index.

### 2.2. Modification of Carbon Black

CB was added to a concentrated citric acid aqueous solution at a ratio of 1:1. This process was developed by variable-frequency ultrasound energy using a 25 mm ultrasonic tip with a 750 W output power and a 50% amplitude. A variable frequency was applied at distinct treatment times (i.e., 15, 30, 45, 60, and 120 min) under an ambient temperature. At the end of the reaction time, the modified carbon black (MCB) nanoparticles were filtered and dried at 80 °C for 24 h. Each sample was identified as shown in Table 1.

### 2.3. Preparation of Nanocomposites

Nylon 6/MCB nanocomposites were obtained using several weight percentages (wt %) of 0, 0.25, 0.5, 1.0, and 2.0 of the MCB nanoparticles and achieved with a reaction time of 60 min. Table 2 shows the amounts used together with their identification. The preparation procedure consisted of mechanically mixing MCB60 nanoparticles and pellets then adding a Thermo Scientific PRIMS TSE-24MC Twin Screw Extruder (Thermo Fisher Scientific, UK) to the mix. This extruder had a diameter of 24 mm and an L/D ratio of 40:1, as well as a plane temperature profile of 210 °C in all zones of the extruder and a 100 rpm velocity of the screws. An adapter of a special design was placed at the end of the extruder for the ultrasonic treatment. A homemade ultrasonic generator produced ultrasonic waves between 15 and 50 kHz with 750 W power.

The nanocomposites obtained were ground and sieved through a 200-mesh sieve. The powder obtained was maintained on the surface of 230-mesh sieves. For performing uric acid adsorption and hemolysis tests, the particle size was between 63 and 74 µm.

### 2.4. X-ray Diffraction (XRD)

X-ray diffractograms of the carbon and nanocomposites samples were observed by a Rigaku X-ray diffractometer, using a CuKα X-ray operated at a current intensity of 25 mA and a voltage of 35 kV. The scanning in the 2ϴ scale was from 15° to 80° with a sample velocity of 0.02°/s.

The crystal size (*L*) for the samples by the Scherrer equation was determined [28].

*L* = 0.89*λ*/*β*cos*θ* (1)

where *λ* is the X-ray wavelength, *β* the full width at half-maximum (FWHM) intensity of the peak, and *θ* is the Bragg’s angle.

### 2.5. Differential Scanning Calorimetry (DSC)

The polymorphic structure, melting temperature, crystallization temperature, and degree of crystallinity of all the samples were determined by means of a calorimeter (Model Q-2000, TA Instruments, USA). The heating and cooling rate were set at 10 °C/min, and high-purity nitrogen (99.999%) with a flow rate of 50 mL/min was used to avoid oxidation of the samples. Between 10 and 15 mg of each sample was tested from 0 to 260 °C. The assays were performed following the ASTM D3418 standard.

The degree of crystallinity was calculated using the following Equation (2)

(2) $$X_{c}\left(\%\right)=\frac{\Delta H_{f}}{\left(1-\;\emptyset \right)\Delta H^{*}}\;\times \;100$$

where $\Delta H_{f}$ is the heat of fusion of every sample and $\Delta H^{*}$ is the heat of fusion of the pure Nylon 6 with 100% crystallinity, equal to 191.064 J/g for the specific case of our polymer [29], and ∅ is the weight fraction of the MCB nanoparticles

### 2.6. Thermogravimetric Analysis (TGA)

Thermogravimetric analysis was employed to study the thermal behavior of the CB and MCB particles using a DuPont Instruments 951 analyzer (Wilmington, NC, USA). The operating conditions comprised a heating rate of 10 °C/min and an air atmosphere with a gas flow of 50 mL/min. The runs of the samples were carried out from 30 to 600 °C in an N_2_ atmosphere. Once 600 °C was reached, the N_2_ atmosphere was changed to O_2_.

### 2.7. Fourier Transform Infrared Spectroscopy (FTIR)

The CB and MCB materials and NY6/MCB60 nanocomposites were characterized by FTIR using a Thermo Nicolet (Model MAGNA 550 GMI, Thermo Fisher Scientific, USA). These analyses were carried out under the following conditions: a wave interval from 4000 to 600 cm^−1^, an Attenuated Total Reflection (ATR) attachment, 100 scans of each sample, and a resolution of 16 cm^−1^. Potassium bromide (KBr, Sigma Aldrich, Mexico) powder is used to prepare KBr pellets for infrared analysis. The powder samples were previously dried in a vacuum oven at 80 °C for 24 h and then ground in a mortar.

### 2.8. Transmission Electron Microscopy (TEM)

The morphology of the CB and MCB was studied via electronic transmission microscopy using TITAN-300kV (FEI, USA) with an objective lens (Type S-TWIN; Cs = 1.3 mm). Micrographs were recorded with a CCD camera near the Scherzer focus. The CB and MCB nanoparticles were dispersed using ultrasound for 5 min, after which they were placed on grids for analysis.

### 2.9. Hemolysis Assay

In accordance with the ASTM E2524-08 standard, and using various concentrations of nanoparticles (i.e., 100, 200, 300, and 400 µg/mL), the hemolysis assay was performed [30]. As a negative control (0% hemolysis) and positive control (100% hemolysis), Dulbecco’s phosphate-buffered saline (DPBS, Sigma Aldrich, Mexico) and polyethyleneglycol (PEG, Sigma Aldrich, Mexico) were used, respectively.

### 2.10. Uric Acid Adsorption

For determining the uric acid adsorption of the CB, MCB60 nanoparticles, and NY6/MCB60 nanocomposites, a calibration curve of uric acid was established (concentrations of uric acid were 80, 100, 120, 140, 160, 180, and 200 mg/L). UV–vis analyses were realized using a Shimadzu UV-1800 model UV–vis spectrometer (Shimadzu, Duisburg, Germany), and the maximum wavelength (λ_max_) was localized at 290 nm. All adsorption experiments were carried out in 250 mL beakers with mechanical agitation of 100 rpm and a temperature of 37 °C. An aliquot of the experiment was taken every 15 min to be analyzed by a UV–vis spectrometer. All experiments and measurements were made in triplicate and statistically analyzed using Analysis of Variances method (ANOVA). Percentages were calculated using Langmuir and Freundlich isotherms for more details are shown in previous work [11]. The removal percentage was calculated by the following equation:

(3) $$\%Removal=\frac{\left(C_{i}-C_{e}\right)}{Ci}\times 100$$

where *C_i_* and *C_e_* are initial and final concentrations, respectively.

## 3. Results and Discussion

### 3.1. CB and MCB Characterization

#### 3.1.1. Thermogravimetric Analysis (TGA)

**Figure 1:**
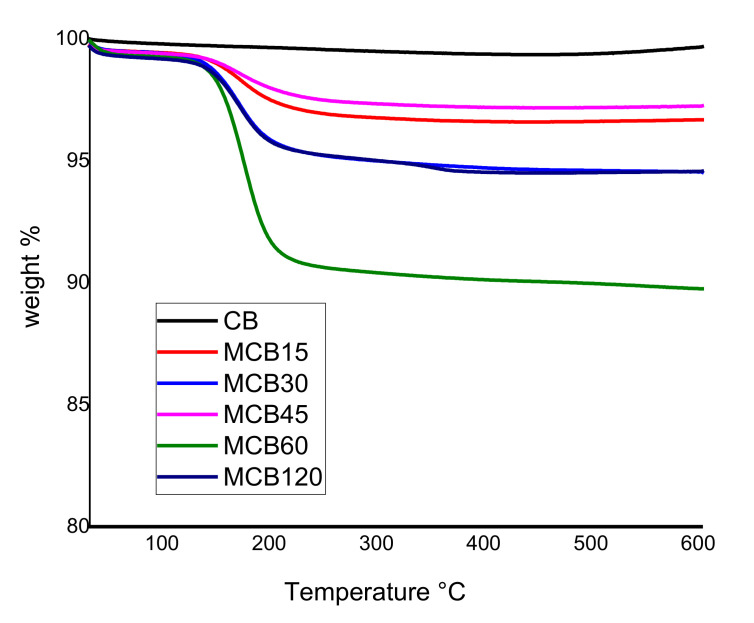
Thermogravimetric analysis of the carbon black and modified carbon black.

Figure 1 shows the results of the TGA of the CB and MCB samples. The results indicate that thermal stability was achieved in unmodified CB, under temperatures ranging from 25 to 150 °C, with a weight loss of only 0.06%, corresponding to the absorbed surface water of material CB. The CB samples modified with citric acid at different reaction times showed less thermal stability when compared to pure CB, along with weight losses that began at 50 °C and remained constant up to 120 °C. Thereafter, the weight loss became more drastic and tended to stabilize at 200 °C. These losses can be attributed to volatiles, whose presence indicates a material with greater hydrophilicity compared to unmodified CB. In 2017, Li et al. reported a similar behavior in calcium carbonate modified with organic acids, including citric acid, and defined that organic acids are thermally degraded and form under temperatures ranging from 120 to 126 °C [31].

The MCB15, MCB30, MCB45, MCB60, and MCB/120 samples showed weight losses of 3.36 ± 0.1%, 5.19 ± 0.2%, 2.64 ± 0.1%, 9.78 ± 0.4%, and 5.24 ± 0.3%, respectively. Sample MCB60 showed the highest weight loss with temperature, showing that a 60 min reaction time is optimal for achieving higher functionalization. Shorter reaction times cause low ultrasound energy and incomplete functionalization, while longer reaction times can lead to the destruction of new functionalities on formed MCB. Similar results were reported Cabello-Alvarado et al. when modifying Multi-Wall Carbon Nanotube (MWCNT) with citric acid, who showed that ultrasound energy causes damage in the structure of MWCNT and a decrease in oxidation [32]. In 2017, Bibi et al. reported that the scope of the surface reaction can be controlled with the modification of sonication time, and this technique can be used to vary the mean length of carbon structures [33].

#### 3.1.2. Fourier Transform Infrared Spectroscopy (FTIR)

FTIR spectra for carbon materials are difficult to obtain, and only in some cases can the presence of functional groups be confirmed by this technique. Generally, when the signals of these functional groups are clearly observed in the spectrum, their presence is accepted; however, there is often disagreement in their assignments, although negative FTIR results do not necessarily confirm the absence of these functional groups.

**Figure 2:**
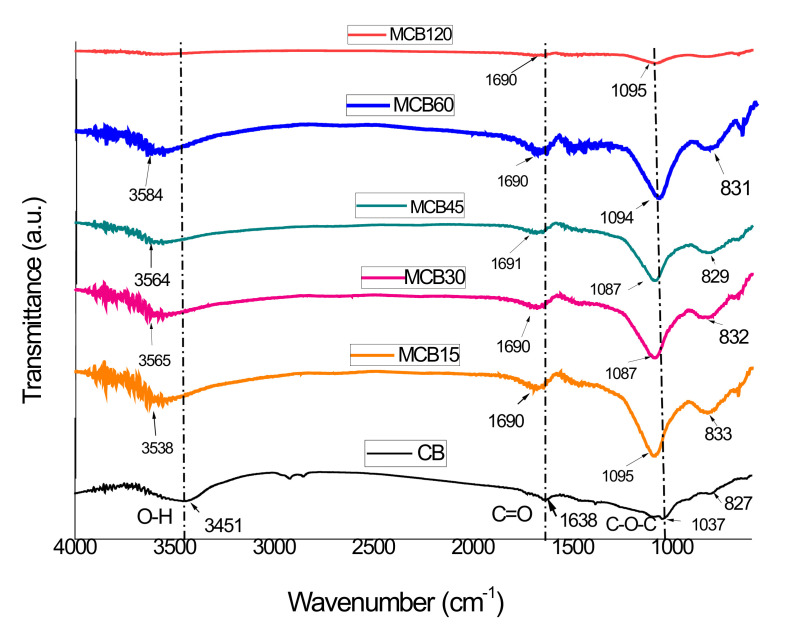
FTIR spectra of the unmodified carbon black and the modified carbon black with citric acid.

The FTIR spectra corresponding to the CB and MBC samples are listed in Figure 2. The CB spectrum shows signals at 3451 cm^−1^ that correspond to the OH stretching vibration from the water absorption or hydroxyl groups (alcohols or phenols) present in the materials. The small bands between 2850 and 2975 cm^−1^ correspond to the C–H stretching, while the small band at 1638 cm^−1^ is ascribed to the C=C stretching vibration present in the material. The wide band centered at 1037 cm^−1^ represents a complex section of the infrared spectra where signals corresponding to the aromatic C–C and C–H plane deformation structures can overlap with signals corresponding to other C–O–C stretching groups [34,35,36]. The wide band at 1037 cm^–1^ is characteristic of carbon structures with a low content of conjugated bonds in surfaces highly oxidized with a high content of C–O–C bonds [37,38]. The results of the FTIR analysis show that CB has no carboxylic acids on its surface, while the presence of phenols and alcohols is not discarded.

The FTIR spectra corresponding to the MCB samples did not show clearly defined signals but rather a new band located at 1690 cm^−1^. This band is within the range of the carbonyl (C=O) adsorption of the carboxylic group (1689–1759 cm^−1^), but its location is different than the band with the carboxylic group of citric acid (1729 cm^−1^). The location of a peak at a lower frequency and another new band located at 1460 cm^−1^ suggest that the –COOH group is involved in an ionic exchange process. Moreover, two new wide and intense bands centered at approximately 1087 cm^−1^ and 829 cm^−1^ were obtained, both of which are attributed to citric acid, which has many signals that can be grouped in the ranges of 430–960 cm^−1^ and 996–1430 cm^−1^ [39]. The elimination of bands corresponding to the C–H vibration (2850–2975 cm^−1^) can be attributed to the presence of a large number of functional groups as a result of the addition of citric acid to the surface of the CB.

#### 3.1.3. X-ray Diffraction (XRD)

**Figure 3:**
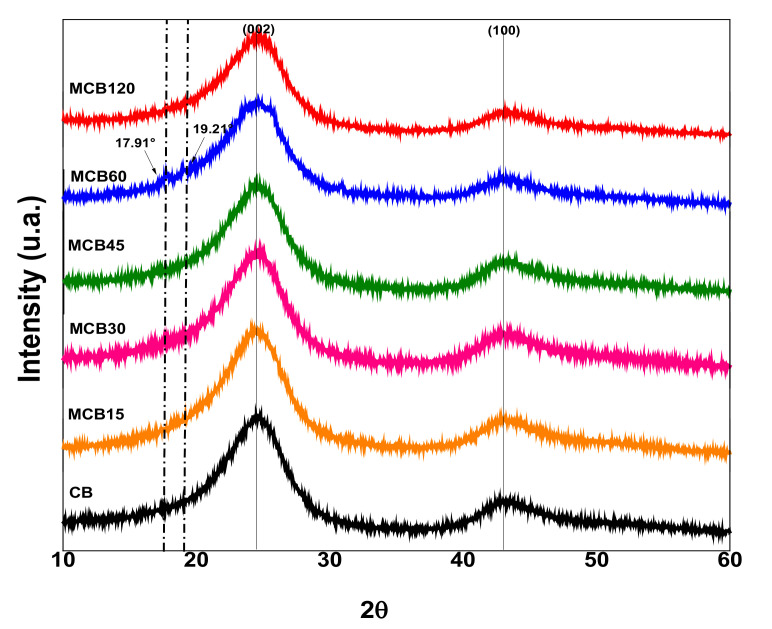
X-ray diffraction patterns of the unmodified carbon black and the modified carbon black with citric acid.

The X-ray diffraction patterns of the unmodified carbon black and the modified carbon black with citric acid are shown in Figure 3. The CB diffractogram shows two diffraction peaks at 24.5° and 43.15° of 2ϴ, assigned to planes (002) and (100) of CB, according to a report by Vicentini et al. [40]. The MCB diffractograms show the diffraction peaks of carbon black, but with slight changes in the location of the peaks, attributed to the addition of functional groups that contain oxygen on the surface of the carbon black, which has been reported by other authors [41,42]. The diffractogram of the MCB60 sample showed two peaks located at 19.21° and 17.91° of 2ϴ, which correspond to the diffraction pattern of CA. These results confirm the greater percentage of functionalization defined by TGA (Figure 1). The XRD results coincide with those reported by Maleki et al., who modified graphene oxide with citric acid [43].

The unmodified CB showed a crystalline structure similar to graphite, and the addition of polar groups to the surface was able to modify this crystalline structure, which can be corroborated by the size of the crystals obtained using the Scherrer equation. The crystal sizes of the pure CB and MCB samples are shown in Table 3. The data listed in Table 3 show that the addition of CA to the surface of CB slightly affected the crystalline structure. The size of the crystals decreased in all samples and was higher at longer reaction times. The surface modification of carbon materials causes different effects in crystalline structures; for example, it has been reported that the modification of CB with a titanate-coupling agent does not modify the crystal size, even with the addition of 0.8–12 wt % [44]. Other reported results conclude that the addition of polar groups to the surface of activated carbon increases crystal size [45].

#### 3.1.4. Transmission Electron Microscopy (TEM)

**Figure 4:**
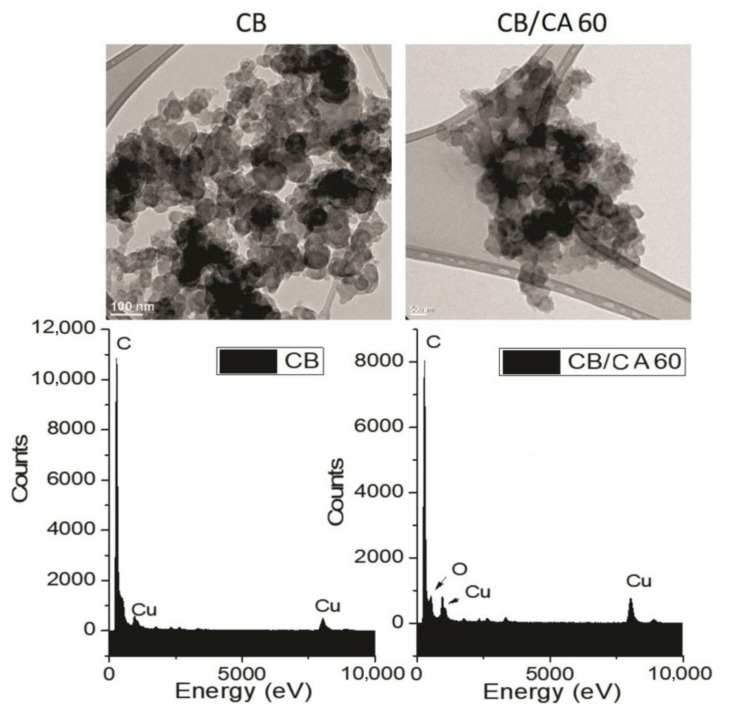
TEM images and EDS spectra of the carbon black and the MCB60 samples.

The TEM technique was used to assess the morphology of the pure CB and MCB60 samples that showed the highest level of modification. Their micrographs are shown in Figure 4. The CB image shows semispherical primary particles with an average size of 20–80 nm. The primary particles coalesced and conformed aggregates, and the strong attraction between the aggregates caused agglomerates of micrometric size between 0.8 and 2.5 µm.

The MCB60 micrograph showed similar characteristics, but the size of the primary particles and the agglomerates was smaller, suggesting that the surface modification of CB favors particle dispersion during the preparation of samples and prevents the formation of agglomerates of a larger size. The semispherical shape of the primary particles showed less resolution due to the presence of an organic layer on the surface. The micrographs obtained in this study are similar to those obtained by Perez et al. when studying the surface chemistry of carbon black Vulcan XC-72R [46].

The energy dispersive X-rays spectroscopy (EDS) spectrum of the CB (Figure 4) shows a signal at a band energy of 279.6 eV corresponding to carbon, as well as two signals located at 984.7 and 8050.9 eV corresponding to the copper grid. The EDS spectrum of the MCB60 sample showed the same signals that the CB did, as well as a new well-defined signal located at a 538.39 eV attributed to the presence of oxygen. Such results coincide with the results reported by Mohan et al. when using carbon black from hydrocarbons soot [38].

#### 3.1.5. Dispersion Stability

CB has several applications in the biological, pharmaceutical, and medical fields, which require the easy dispersion of nanomaterials in different polar and nonpolar solvents [38]. Table 4 shows images of the dispersion tests of the modified and unmodified particles after 48 h of sonication. When comparing the images, it is noted that the CB showed precipitates, while the solutions of MCB showed better stability and were easily dispersed in polar solvents such as distilled water and ethanol. The nanoparticles were homogeneously dispersed and remained in the suspension after 48 h. This behavior could be the result of the formation of interactions of hydrogen bonds between functional groups of MCB (carboxylic) and of ethanol and distilled water (hydroxyls) solvents. Some authors have reported similar behavior after modifying carbon nanoparticles [47,48].

### 3.2. Characterization of NY6/MCB60 Nanocomposites

#### 3.2.1. Fourier Transform Infrared Spectroscopy (FTIR)

**Figure 5:**
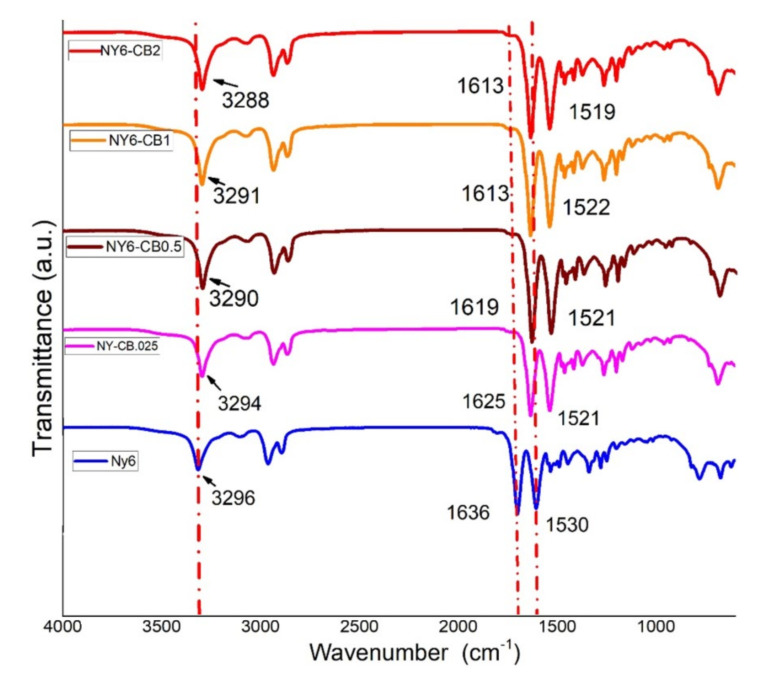
FTIR spectra of the Nylon 6 and NY6/MCB60 nanocomposites.

Figure 5 shows the FTIR spectra of the Nylon 6 and NY6/MCB60 nanocomposites. The Nylon 6 spectrum shows a sharp and very strong band at 3296 cm^−1^ that it is assigned to NH stretching, as well as two bands located at 2934 and 2859 cm^−1^ assigned to the asymmetric and symmetric stretching vibrations of the CH_2_ groups. Other strong bands were detected at 1636 and 1530 cm^−1^, the first of which is assigned to amide I, which has the main contribution of C=O stretching [49,50]. The second band is assigned to amide II, to which NH deformation mainly contributes.

The FTIR spectra of the nanocomposites are very similar to those of Nylon 6, but the NH stretching, C=O stretching, and NH deformation of the bands are shifted to a lower frequency. The shift is higher in terms of the amount the MCB increased. This behavior is explained by the presence of hydrogen bonds between the functional groups of Nylon (C=O and –NH) and MCB (COOH) [51]. The location of C=O and NH bands at 1636 and 1530 cm^−1^, respectively, indicating the presence of the C=O---HN- type hydrogen bonds in the Nylon 6.

In the nanocomposites, the adsorption corresponding to NH deformation is shifted to a lower wavelength (1519 cm^−1^), suggesting the presence of C=O-OH---NH- type hydrogen bonds, indicating a strong interaction between MCB and Nylon 6 that might have significant effects on crystallinity and mechanical properties.

#### 3.2.2. Thermogravimetric Analysis (TGA)

**Figure 6:**
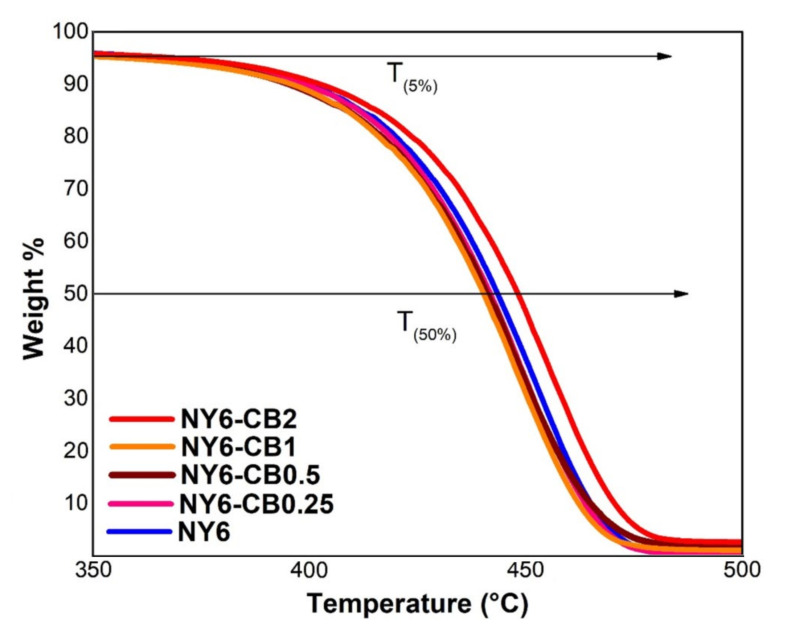
Thermogravimetric analysis of the Nylon 6 and NY6/MCB60 nanocomposites.

TGA of the Nylon 6 and the NY6/MCB60 nanocomposites at concentrations of CB in the range of 0.25–2.0 wt % is shown in Figure 6, in which it can be noted that all samples had similar thermal behavior. The first weight loss was found in the temperature range of 36–333 °C, which is related to water adsorbed on the surface of the materials. The second weight loss occurred in a temperature range of 340–526 °C, which is attributed to the thermal degradation of the Nylon 6 [52,53].

The amount of residues at 550 °C of the Nylon 6 and the NY6/MCB60 nanocomposites are reported in Table 5. The obtained results are similar to the MCB60 content used in the original formula. The TGA results confirm that the addition of MCB60 particles to the Nylon 6 matrix does not improve thermal stability and that higher stability is only achieved when the content of MCB60 is of 2 wt %.

#### 3.2.3. Differential Scanning Calorimetry (DSC)

The melting temperature (T_m_), crystallization temperature (T_c_), glass transition temperature (T_g_), and degree of crystallinity (X_c_) for Nylon 6 and the nanocomposites were confirmed by DSC analysis. The results are shown in Table 6. The addition of MCB60 to the Nylon 6 matrix caused major changes in the thermal properties. The T_m_ did not show significant changes in general, and the values of nanocomposites were slightly lower in comparison to the T_m_ of Nylon 6. The data in the literature were controversial regarding this effect and report that values of T_m_ can increase or decrease with the addition of loads of MCB60 [27,54]. The T_g_ and T_c_ increased significantly even with a low content of MCB60. These results indicate that MCB60 particles restrict the mobility of Nylon 6 chains, and the crystallization process of nanocomposites begins faster in comparison to pure Nylon 6 [55]. This behavior is associated with the effect of MCB60 as a support of nucleation in the Nylon 6 matrix, and similar results have been reported in Nylon 6/CB nanocomposites [27,54].

Other evidence that supports the nucleating effect of MCB60 particles is the increase in X_c_ to the extent that the content of MCB60 increased, and the NY6/MCB60-2.0 nanocomposite with a load percentage of 2 wt % showed a higher level of crystallization (51.0%). These findings suggest that the number of crystallizable Nylon 6 chains increased because the presence of MCB60 generated nucleation sites in Nylon 6.

Therefore, the addition of MCB60 nanoparticles to the Nylon 6 matrix affects the thermal and mechanical properties of the nanocomposite, although the extent to which depends on several factors, the main factors being the characteristics of the NPs and the concentration used.

#### 3.2.4. Hemolysis Assay

**Figure 7:**
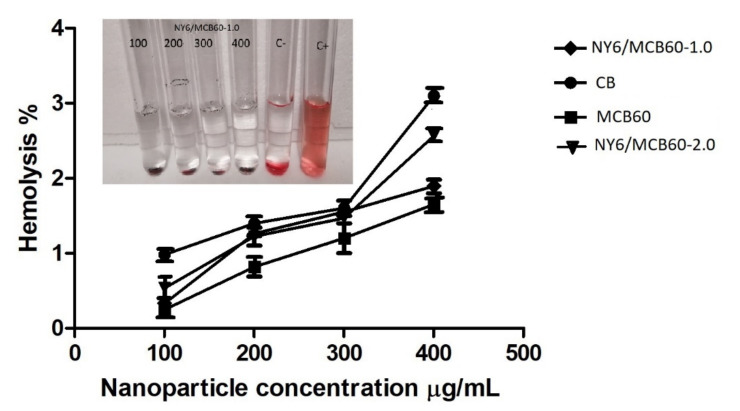
Hemolysis study results of CB, MCB60 and NY6/MCB60 nanocomposites (concentrations of 100, 200, 300, and 400 µg/mL; C-, negative control; C+, positive control).

To assess the impact of MCB on erythrocyte, a hemolysis test was conducted in accordance with the ASTM E2524-08 standard. The materials assessed under this standard were particles of carbon black, carbon black modified with citric acid, and Nylon 6 nanocomposites with an MCB60 content of 1 and 2 wt %. As an important note, the Nylon 6 was not assessed since this material has no nanoparticles. Figure 7 shows a graph from the hemolysis assay test, and in the picture can be seen the concentrations different (100, 200, 300, and 400 µg/mL) and C-negative and C-positive controls, which were tested for the hemolysis assay of NY6/MCB60-1.0. The percentage observed in all concentrations tested was low compared with the positive control and did not note the cellular lysis of erythrocytes.

The unmodified carbon black showed hemolysis of 3% and the same material modified with citric acid showed a percentage of hemolysis of 1.6%. These results indicate that CB and their derivatives have no hemolytic potential and coincide with those reported by others [56,57]. The NY6/MCB60-1.0 and NY6/MCB60-2.0 nanocomposites (with 1 and 2 wt % of MCB, respectively) showed low percentages of hemolysis (1.8% and 2.4%). This study suggests that the combination of the polar polymer as Nylon 6 with MCB leads to materials with low hemolytic potential, meaning that they have excellent blood compatibility. Similar conclusions were reported by Pajnic et al. when studying the effect of carbon black in biological membranes such as erythrocytes, who concluded that there is no damage by the presence of agglomerates of carbon black [57].

#### 3.2.5. Uric Acid Adsorption

**Figure 8:**
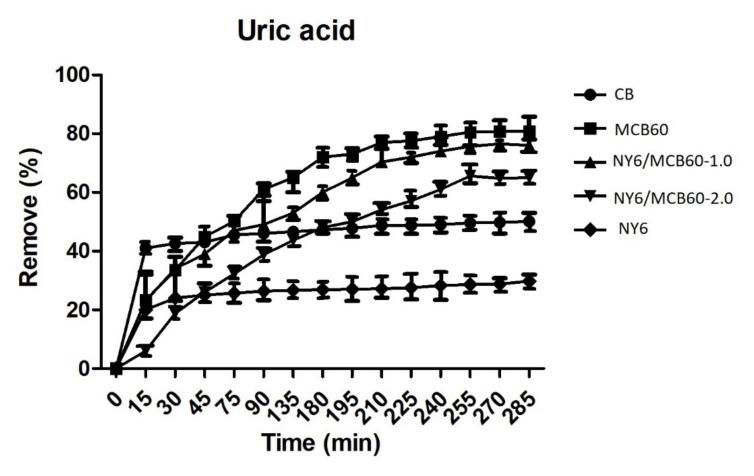
Removal percentage of uric acid of the Nylon 6, CB, and derivatives.

The uric acid adsorption ability of the materials used in this study was assessed in an aqueous solution of known concentration. The results are listed in Figure 8.

The Nylon 6 and CB pure materials showed lower levels of adsorption, and the surface modification of CB with citric acid (MCB60) led to higher adsorption (82%). Nylon 6 nanocomposites with MCB60 retained good uric acid adsorption properties, while the NY6/MCB60-1.0 and NY6/MCB60-2.0 nanocomposites showed high rates of uric acid removal (78% and 65%), respectively). The nanocomposites results confirm that the MCB nanoparticles were properly dispersed in the Nylon 6 matrix, which can be attributed to the ultrasound-assisted extrusion process used in the synthesis nanocomposites.

The new NY6-MCB60 materials are an excellent alternative for use in hemodialysis treatments, since they can be manufactured at a great scale and low cost with no hemolytic potential. The MCB60 percentages used in the assessment were low. A report from the literature concluded that modified graphene nanoplatelets (MGNP) with amines can achieve the removal of 95% uric acid [10]. This result is extraordinary; however, in order to know the real potential of this material, more studies should be carried out where MGNP are added to different polymers and evaluated in different nanocomposites. Carbon black is a low-cost material when compared to graphene nanoplatelets, and the surface functionalization with citric acid affords excellent adsorption properties that are retained when combined with polymers (its efficiency only decreases by 4%). Uric acid cleaning in a hemodialysis session is considered low when the percentage of removal is less than 65%, moderate efficiency between 65% and 80%, and good efficiency higher than 80% [58]. Therefore, the new materials studied in the present work can be considered of moderate and good efficiency.

**Figure 9:**
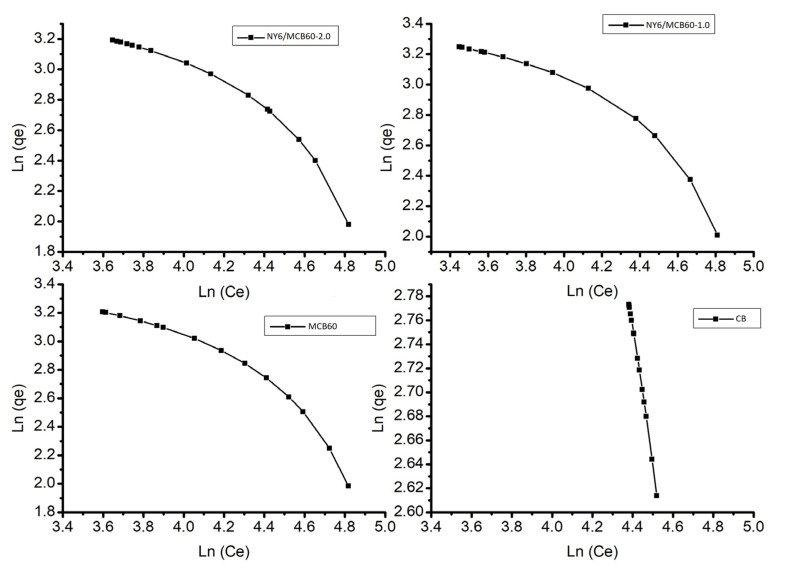
Langmuir and Freundlich model of adsorption for uric acid of the Nylon 6, CB, and derivatives.

Figure 9 shows the results of the Langmuir and Freundlich isotherms, where all samples have heterogeneous adsorption behavior, since all of the materials show a multilayer-type Freundlich isotherm. This isotherm assumes that the concentration in the surface of the adsorbent increases to the same extent that the adsorbate concentration increases [59]. Table 7 lists the data of constants and correlation coefficients.

#### 3.2.6. FTIR Spectra of the Nanocomposite before and after Uric Acid Adsorption

**Figure 10:**
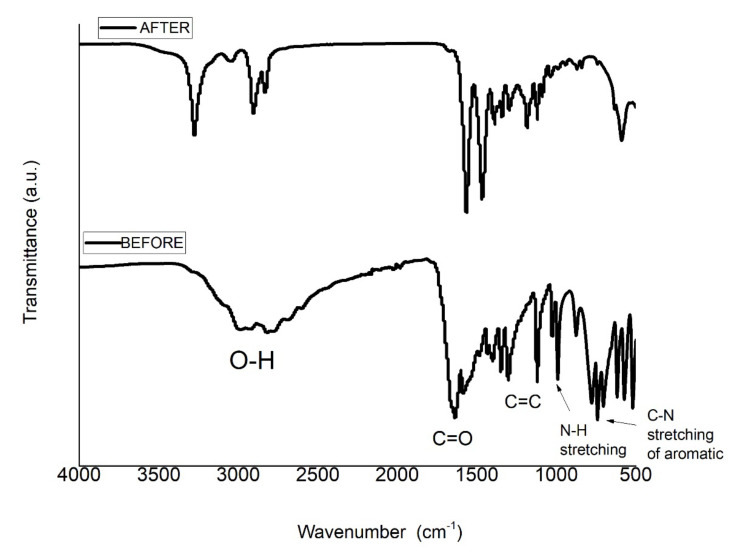
FTIR spectra of the NY6/MCB60-1.0 before and after uric acid adsorption.

The adsorption of uric acid with NY6/MCB60-1.0 nanocomposite was confirmed using FTIR and the spectra of the nanocomposite before and after uric acid adsorption are shown in Figure 10. The NY6/MCB60-1.0 spectrum before adsorption only showed the characteristic signals of the nanocomposite (see Figure 5), while the spectrum obtained after the adsorption showed two new bands located at 2983 and 2806 cm^−1^ corresponding to the N–H stretching, one strong band at 1633 cm^−1^ assigned to the carbonyl group, one band at 1583 cm^−1^ was assigned to C=N, and one band at 742 cm^−1^ belonging to the C–N bond. The FTIR confirmed the presence of bands characteristic of uric acid, while some reports have confirmed the listed evidence in the present study [60,61].

## 4. Conclusions

The surface modification of CB with citric acid using variable-frequency ultrasound leads to a new material (MCB60) with excellent adsorption of uric acid and low hemolytic potential. NY6/MCB60 nanocomposites were obtained using the ultrasound-assisted melt-extrusion method. The new nanocomposites increased the T_c_, T_g_, and X_c_ parameters with respect to pure Nylon 6, suggesting good dispersion of the MCB60 particles and a nucleating effect in the Nylon 6 matrix as a result of good interface interaction between MCB60 and Nylon 6.

Likewise, the NY6/MCB60 nanocomposites showed good adsorption of uric acid and low hemolytic potential, achieving values of 78% and 1.8%, respectively. The method used to prepare the nanocomposites is considered proper for the environment, easily scalable, and economical. These characteristics suggest that NY6/MCB60 nanocomposites can be considered excellent alternatives for manufacturing polymeric membranes for the removal of uric acid from the blood.

## Figures and Tables

**Table 1:** Identification of the samples according to the treatment time.

Sample	Reaction Time (min)	Modifier Acid
CB	0	None
MCB15	15	Citric acid
MCB30	30	Citric acid
MCB45	45	Citric acid
MCB60	60	Citric acid
MCB120	120	Citric acid

**Table 2:** Quantity and Identification of the nanocomposites used.

Nanocomposite	Polymer Content (g)	MCB60 (g)	wt % of the Additive
NY6	400	0	0
NY6/MCB60-0.25	399	1	0.25
NY6/MCB60-0.5	398	2	0.5
NY6/MCB60-1.0	396	4	1.0
NY6/MCB60-2.0	392	8	2.0

**Table 3:** The crystal size of carbon black (CB) modified with citric acid to different reaction times.

Sample	Crystal Size (nm)
CB	1.4501
MCB15	1.3800
MCB30	1.4030
MCB45	1.4258
MCB60	1.2659
MCB120	1.3102

**Table 4:** Stability of the dispersion of the carbon black and modified carbon black with citric acid in water and ethanol.

Solvent	CB	MCB15	MCB30	MCB45	MCB60	MCB120
Water	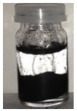	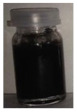	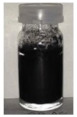	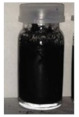	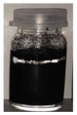	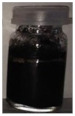
Ethanol	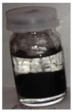	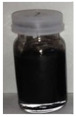	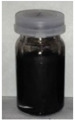	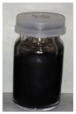	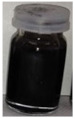	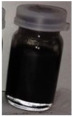

**Table 5:** Amount of residues at 550 °C of the Nylon 6 and NY6/MCB60 nanocomposites.

Sample	Amount of Residue at 550 °C (%)
NY6	0
NY6/MCB60-0.25	0.25
NY6/MCB60-0.5	0.69
NY6/MCB60-1.0	1.04
NY6/MCB60-2.0	2.29

**Table 6:** Differential scanning calorimetry (DSC) data for the Nylon 6 and NY6/MCB60 nanocomposites.

Sample	T_m_ (°C)	T_c_ (°C)	Tg (°C)	X_c_ (%)
NY6	222.43	174.81	41.91	31.35
NY6/MCB60-0.25	221.06	192.36	43.01	48.27
NY6/MCB60-0.5	220.93	193.36	-	47.39
NY6/MCB60-1.0	221.23	193.39	46.98	49.89
NY6/MCB60-2.0	220.76	193.95	-	51.09

**Table 7:** Parameters of isotherm for uric acid adsorption, according to the Langmuir and Freundlich approach.

Sample	Langmuir			Freundlich		
	k	q_max_	R^2^	n	kf	R^2^
CB	0.14	6.66	0.9980	1.15	7.81	0.9982
MCB60	0.11	2.96	0.8735	0.73	5.86	0.9010
NY6/MCB60-1.0	0.14	4.80	0.8737	0.84	6.33	0.9044
NY6/MCB60-2.0	0.14	4.85	0.8877	0.84	6.33	0.9022
